# Defining a Relationship Between Postoperative Antibiotic Use and Wound Complications in the Setting of an Uncomplicated Laparoscopic Appendectomy

**DOI:** 10.7759/cureus.40603

**Published:** 2023-06-18

**Authors:** Matthew Sturdivant, Patrick Downs, Jorge Lara-Gutierrez, Majed Maalouf, Christopher Esper, William Gilleland, Jon Henwood, Christopher Myers, Pablo Giuseppucci

**Affiliations:** 1 General Surgery, University of Pittsburgh Medical Center (UPMC) Horizon, Hermitage, USA

**Keywords:** laparascopic surgery, wound infections, general and laparoscopic surgery, post-op complications, s: appendectomy

## Abstract

Introduction: Appendicitis is a very common diagnosis that surgeons manage daily. Some surgeons are still giving antibiotics to patients suffering from uncomplicated appendicitis in the postoperative setting, despite an abundance of evidence to support a single preoperative dose of antibiotics. In this paper, we will describe the management of post-operative antibiotics at our institutions following uncomplicated appendicitis with regard to the use of antibiotics in the post-operative setting.

Methods: A retrospective chart review was performed analyzing post-operative antibiotic use and postoperative complications in 179 patients undergoing laparoscopic appendectomy for uncomplicated appendicitis. We retrospectively examined the patients to change our future practices as we perform appendectomies routinely, and there is practice variation at our centers. Current Procedural Terminology (CPT) codes for ‘laparoscopic appendectomy’ were used to identify the patients within our inclusion criteria. Twenty-four patients were excluded from the analysis as they had complicated appendicitis or met other exclusion criteria. We only studied the patient with uncomplicated appendicitis, as those with complicated appendicitis have a different clinical course that involves post-operative antibiotic administration or prolonged antibiotic administration with or without drain placement. Both arms of the study were homogeneous regarding patient characteristics. An independent test of the development of wound infection for those patients receiving post-operative antibiotics versus those not receiving post-operative antibiotics was conducted using the SPC XL 2010 Microsoft Excel (Redmond, USA) add-in. A p-value of <0.05 was considered statistically significant. This included the odds ratio for the development of complications.

Results: There was no difference in the risk of infection rate in patients given post-operative antibiotics; however, given the odds ratio of 6.53, there is an association between an increased wound infection rate and patients who received post-operative antibiotics.

Discussion: An appendectomy is a standard surgical procedure for acute appendicitis. The guidelines for using pre-operative antibiotics in uncomplicated appendicitis are well established; however, there is no specific recommendation on whether to continue antibiotics post-operatively. However, there is significant provider variability on this topic. Antibiotic use post-operatively in clean-contaminated cases, such as uncomplicated acute appendicitis, has been associated with higher risks of surgical site infections.

Conclusion: The use of antibiotics post-operatively may not be indicated for uncomplicated laparoscopic appendectomy and may increase wound infections. A large-scale study including a larger population and extending it to other hospitals may increase statistical significance and help guide physician management.

## Introduction

In 1544, Jean Fernel first described acute inflammation of the vermiform appendix [1]. This was not the first description, however. An Egyptian mummy demonstrating adhesions in the right lower quadrant was thought to be secondary to old acute appendicitis from the Byzantine era that had previously been described. [1]. Dr. Reginald H. Fitz first coined the phrase appendicitis in 1886 [1]. The diagnosis of acute appendicitis remains one of the most common indications for emergent surgery worldwide. Appendicitis is described as either complicated or uncomplicated. Uncomplicated appendicitis is defined as acute appendicitis that presents without signs of perforation, such as an inflammatory mass, phlegmon, abscess [2], or other localized inflammatory findings in the appendix. Post-operative complications from appendicitis include staple line leaks, abscesses, wound infections, and bleeding. 

The treatment of choice for uncomplicated appendicitis is appendectomy, as has been proven historically through various other attempted and unsuccessful treatment modalities [1]. Pre-operative prophylactic antibiotic use is imperative to prevent intra-abdominal abscesses, wound infections, and other infectious complications in the post-operative setting [3]. In addition, prophylactic antibiotic use is essential in surgery for procedures that are not considered clean procedures, as the risk of infectious complications is much higher without prophylactic antibiotic administration [4]. The use of perioperative antibiotics for prophylaxis has been heavily studied in the surgical literature. The Surgical Care Improvement Projects (SCIP) criteria section for preoperative antibiotics is a useful tool to guide therapy [5]. However, post-operative antibiotics after an uncomplicated laparoscopic appendectomy are based on surgeon preference and continue to be given. The surgeon’s preference to give antibiotics to patients with acute uncomplicated appendicitis was not based on a protocol or evidence-based medicine. This paper will report a two-year experience regarding post-operative antibiotic use in uncomplicated laparoscopic appendectomy by several surgeons at two community-based teaching hospitals. 

## Materials and methods

**Figure 1:**
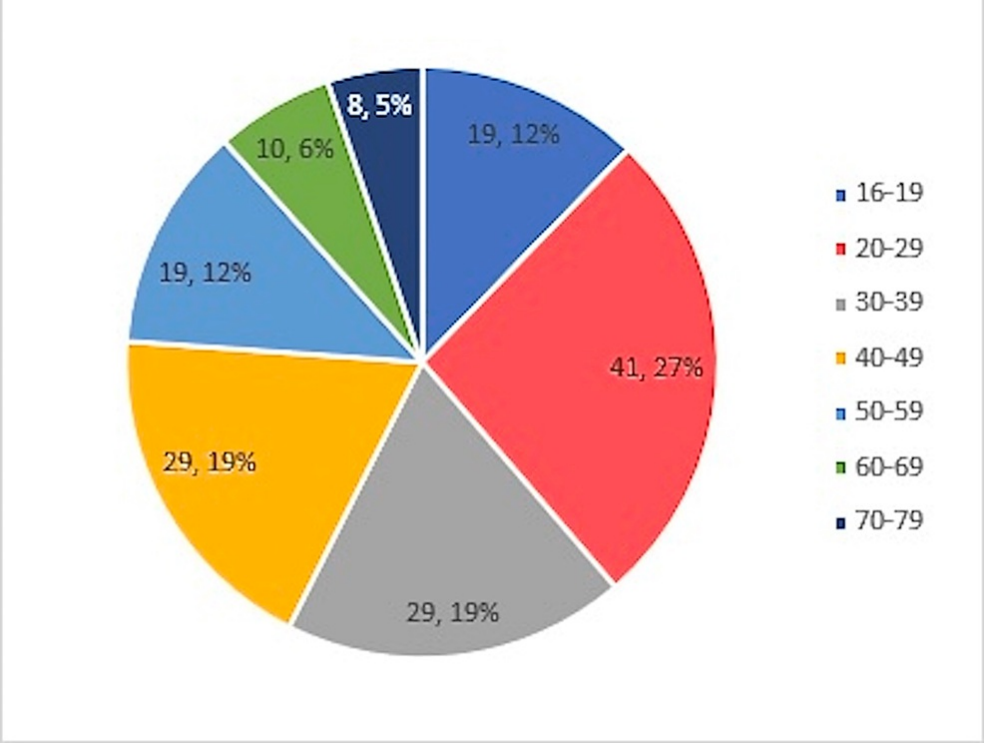
Ages of included patients

The UPMC IRB approved this quality and safety improvement project before any review of patient records. The review was performed at two community-based hospitals in Western Pennsylvania: UPMC Horizon-Farrell and UPMC Jameson. In addition, we accumulated peri-operative and operative data from electronic medical record reviews from six different UPMC-employed general surgeons. All patients with CPT code 44970 (laparoscopy, appendectomy) were retrospectively identified for the timeframe of January 1, 2018, through January 31, 2020. A total of 179 patients were treated. The inclusion criteria were adult patients aged 16 and above (Figure 1) with acute appendicitis given pre-operative antibiotics. In addition, the patients had to have undergone an uncomplicated laparoscopic appendectomy at one of two facilities during the desired study period and participated in the post-operative follow-up. The majority of patients were in the age bracket of 20-29 years old, a common age group for this problem. The exclusion criteria were patients younger than 16 years of age, those with complicated appendicitis, pregnant females, incidental appendectomies performed during other procedures, and patients with appendiceal cancer and mucocele findings on surgical pathology. 

The operative techniques were similar among the surgeons involved in the review but were not standardized. Foley catheters were placed after induction of anesthesia under sterile conditions and were removed after the surgery. The ports were placed in a standard fashion, with the left lower quadrant, peri-umbilical, and suprapubic standard laparoscopic access areas for this procedure. Some surgeons used the open Hassan technique for umbilical access, while others used a Veress needle to establish access to establish pneumoperitoneum. The appendix was identified and then mobilized. Dissection and stapling of the appendix also varied between the surgeons. Some surgeons start by opening a window at the base of the mesoappendix, while others dissect the distal mesentery and continue the mesenteric dissection proximally to the base of the appendix. A varied number of staple loads were used to staple and transect the appendix and the mesoappendix. In all cases, an endo catch bag was used to remove the appendix from the peritoneal cavity. The right lower quadrant and pelvis were examined for bleeding, purulent fluid, and serous fluid, and finally, both areas were irrigated. All port sites were closed at the skin level. The fascia of the 12mm ports was also closed, depending on the post-operative diameter of the fascial defect, with sutures in a typical figure-of-eight fashion. The suture used for the closure was variable and dependent on the surgeon’s preference. Access, dissection, and stapling also depended on the patient's anatomy and visualization.

Several different antibiotics were given pre-operatively and post-operatively and were based on surgeon preference. 

A total of 24 patients were excluded from the study: perforation (8), appendiceal mucocele (1), abdominal abscess (1), incidental appendectomy (1), isolated urinary tract infection (3), loss to follow-up (2), non-infectious serous/bloody drainage (2), hypotension (1), diarrhea/abdominal pain (1), dizziness/headache (1), symptoms of biliary colic (1), and nausea/vomiting with abdominal pain (1). 

An independent test of the development of wound infection for those patients receiving post-operative antibiotics versus those not receiving post-operative antibiotics was conducted using the SPC XL 2010 Microsoft Excel add-in (SPC XL 2010©, version 2.50.0600, DBA SigmaZone.com & Air Academy Associates, LLC, Orlando, FL, USA). A p-value of <0.05 was considered statistically significant. 

Operative findings were identified through a thorough chart review. Six (6) categories were selected: no fluid or necrosis, serous fluid, inflammatory fluid, purulent fluid, necrosis, and other cyst/tumor. The calculation of p-values was completed using the Microsoft Excel chi-square test (chi-square test) for all operative finding categories. 

The analysis included an odds ratio of patients who developed wound infections after receiving post-operative antibiotics for patients who developed wound infections without receiving post-operative antibiotics. 

## Results

Our patient population included patients aged 16 to 78, with a median age of 34.5 years. In addition, we examined the patients' electronic medical records postoperatively to evaluate problems in the office or emergency department or lack thereof. Three patients (1.9%) presented to the emergency department with infectious complications or complaints. Some of the postoperative complaints were pertinent to the review, while others were not related to the surgery and thus were not included.

After a thorough chart review and analysis of statistics, our data was presented. Out of the 155 patients, 61 (39.3%) received postoperative antibiotics despite having an uncomplicated laparoscopic appendectomy performed for uncomplicated appendicitis. This left 94 patients (60.6%) without antibiotics in their postoperative course. The route, duration of administration, and type of antibiotics varied among the patients (Table 1 ). 

**Table 1:** Postoperative antibiotic doses given during the study and associated costs N/a = no institutional data available. Antibiotic combination note: one patient received cefoxitin and unasyn (one dose each), and two patients received ciprofloxacin and flagyl (one dose of ciprofloxacin and two doses of flagyl).

Antibiotic	Patients without wound infection (N = 150)	Patients who developed wound infection (N = 5)	Cost per dose ($)	Doses administered post-operatively	Total Cost ($)
Cefoxitin	40	2	3.76	97	364.72
Pipracillin, tazobactam	12	1	12.3	28	344.4
Ampicillin, sulbactam	0	1	n/a	0	n/a
Cefazolin	1	0	3.22	2	6.44
Ciprofloxacin	2	0	2.17	2	4.34
Metronidazole	2	0	1.03	4	2.06
Clindamycin	2	0	4.45	3	13.4

**Figure 2:**
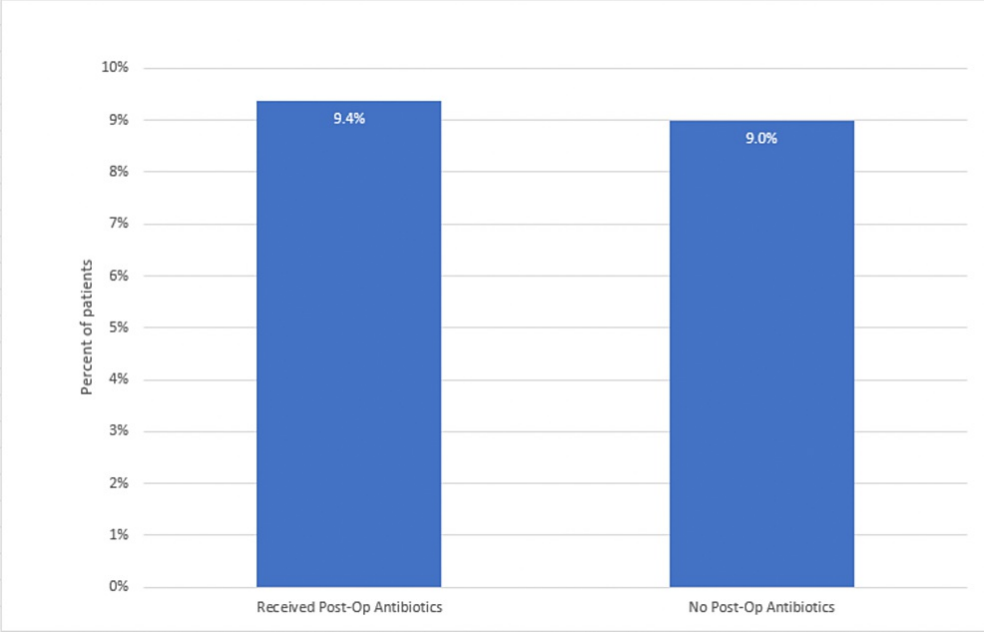
Percent of patients with any type of postoperative complication with or without administration of postoperative antibiotics (p-value = 0.0586)

**Figure 3:**
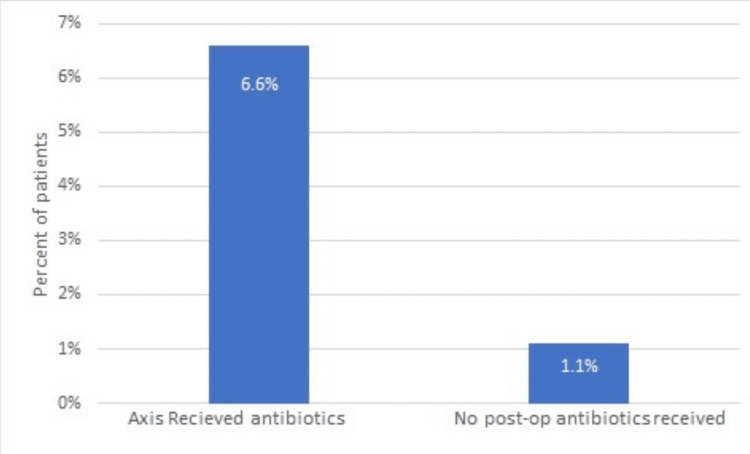
Percent of patients with postoperative wound infections that received or did not receive postoperative antibiotics (p-value = 1)

Overall, 150 patients (96.7%) had no postoperative wound complications, and five patients (3.2%) had postoperative complications. Nine patients had complications in the antibiotic arm, and six patients had complications in the no-antibiotic arm. Infections and postoperative complications were identified during physical exams and on review of electronic medical records. However, no cultures were taken of the wound infections (Figures 2, 3). 

There were several different antibiotics given during the perioperative period. Patient allergies and surgeon preference drove the choice of antibiotics. Cefoxitin was the antibiotic given most frequently. 

In total, there were eight patients (13.1%) in the postoperative antibiotic group that developed significant postoperative complications. Of these, four patients (50%) developed wound infections. However, three patients (3.2%) in the no postoperative antibiotic group developed postoperative complications; of these, only one (33%) developed a wound infection. The Chi-square analysis revealed no statistically significant difference (p = 0.5487; the accepted alpha standard of p <0.05) between postoperative complications if antibiotics were given in the pre-operative setting for acute uncomplicated appendicitis. This shows that the administration of antibiotics for acute uncomplicated appendicitis in the postoperative setting is not inferior to withholding them; however, it appears it is unnecessary. Table 2 shows details of postoperative antibiotic use.

**Table 2:** Patient demographics with descriptions of operative findings and data analysis with findings of wound infections and without wound infections Categorical data p-values are identified by the chi square test in Microsoft Excel.

		Patients without wound infection (N = 150)	Patients who developed wound infection (N = 5)	P-value
	Mean Age (years +/- standard deviation)	37.66 +/- 16.18	36.4 +/- 10.59	-
Postoperative antibiotic use	Not received	93 (62%)	1 (20%)	-
	Received	57 (38%)	4 (80%)	-
	No fluid or necrotic appendix	121 (78.06%)	4 (2.58%)	0.987
	Serous fluid	8 (5.16%)	0 (0.0%)	0.606
	Inflammatory fluid	8 (5.16%)	0 (0.0%)	0.606
	Purulent fluid	8 (5.16%)	0 (0.0%)	0.181
	Necrotic appendix	3 (1.94%)	0 (0.0%)	0.752
	Other cyst or tumor	2 (1.29%)		0.796
	Overall p-value			0.0586

The odds ratio between the two arms was calculated to be 6.53, demonstrating that patients are more than six times more likely to get an infection in the postoperative setting if antibiotics are also administered in the postoperative setting (Table 3). 

**Table 3:** Overall operative outcomes with odds ratio

	Patients who received PO antibiotics (N = 61)	Patients who did not receive PO antibiotics (N = 94)	
No complications	53 (86.89%)	91 (96.81%)	
Wound infection	4 (6.56%)	1 (1.06%)	
Dehiscence	0 (0.0%)	1 (1.06%)	
Return to surgery	1 (1.64%)	0 (0.0%)	
Readmission	1 (1.64%)	1 (1.06%)	
Other complications	2 (3.28%)	0 (0.0%)	
Odds ratio	-	-	6.53

## Discussion

Our data suggest that postoperative antibiotics, while not statistically significant, may increase the risk of wound-related and infectious postoperative complications after an uncomplicated laparoscopic appendectomy. Antibiotic side effects have been demonstrated throughout clinical practice, and decreasing their prevalence in surgical patients is important. Surgical literature further demonstrates only a need for prophylactic pre-operative antibiotics in the setting of uncomplicated acute appendicitis [6-10]. 

Children diagnosed with uncomplicated appendicitis should receive a single dose of preoperative antibiotics. Preoperative antibiotics reduced the incidence of intra-abdominal abscesses and wound infections [3]. 

For adults with uncomplicated appendicitis, the obligatory use of prophylactic antibiotics prevents intra-abdominal and wound infections. In a review by Douglas Smink and David Soybel, they found that a single dose of antibiotics is adequate for preventing wound infection and intra-abdominal abscess [4]. Brian A. Coakley reported that patients who received postoperative antibiotics had a longer length of stay and similar wound infections than those that did not receive postoperative antibiotics [6]. A 10-year study by Dinhlkim Le that included over 500 patients suggested no clinical benefit to the use of postoperative antibiotics. [7]. Similarly, a study by the Department of Surgery from Edinburgh showed that postoperative antibiotics did not alter intra-abdominal abscesses [8]. Another study performed by the Department of Surgery at Mount Sinai Medical Center found that the use of antibiotics correlated with a significantly longer postoperative length of stay and did not reduce the risk of developing superficial surgical site infections, organ space infections, or urinary tract infections [6]. Hussain described in their study of 370 patients that a single dose of antibiotics was sufficient to reduce surgical site infections. There is no difference compared with patients who received postoperative antibiotics. Regarding guidelines, the European Association for Endoscopic Surgery (EAES) Consensus Conference 2015 and an additional study mention that there is no evidence supporting the use of postoperative antibiotics [9,11]. Another study involving academic teaching hospitals, community teaching hospitals, and community non-teaching hospitals with a total of 1975 patients found that postoperative antibiotics are not associated with a reduction in complications [10]. 

The odds ratio of 6.53 further demonstrates the association that antibiotics should not be given in postoperative acute uncomplicated appendicitis, as proven by a large amount of literature. This increased complication rate seen with postoperative antibiotics could be due to varying clinical surgeon sensitivities to the severity of appendicitis. With six different surgeons, each having their own threshold for postoperative antibiotics, some may have given a postoperative course of antibiotics if there was significant surrounding inflammation and possible reactive fluid, despite not meeting the criteria for complicated appendicitis. If this were the case, then those patients who received postoperative antibiotics could have had a greater amount of inflammation and micro-perforation and, thus, a greater risk for infection and complications. It was estimated that a total of $700 worth of antibiotics were given to our patients. Given that our sample size was small, if this were applied to a larger population, this could represent an area of significant healthcare and hospital cost savings. 

During our analysis, four (6.56%) patients who received antibiotics had postoperative wound infections, and only one (1.64%) patient who did not receive antibiotics had wound infections. 

Our study was limited by the small sample of patients, the variability of antibiotic choice and duration, and the variable techniques of appendectomy. Performing a similar retrospective study including more patients and surgeons may provide a higher-powered review if this trend of giving postoperative antibiotics against the literature is part of other surgeons’ practices. This would provide more substantial evidence and may lead to a scientific-based recommendation regarding antibiotics postoperatively. Based on the presented literature and our own data, our practices have aligned with the previously published evidence, and our patients are no longer receiving postoperative antibiotics for acute uncomplicated appendicitis. 

## Conclusions

Our data suggest that antibiotic use in the postoperative setting after an uncomplicated laparoscopic appendectomy is not beneficial and may be associated with a higher wound infection rate. This retrospective review was performed as there is still practice variation with regard to post-operative antibiotics, despite an abundance of literature supporting not using them in this setting. With all of the literature available describing that the use of antibiotics in this setting is either not helpful or even hurtful to the patient, our practice has changed to align with the robust evidence. 
